# Mortality and guideline-concordant care for older patients with schizophrenia: a retrospective longitudinal study

**DOI:** 10.1186/1741-7015-10-147

**Published:** 2012-11-26

**Authors:** Jack Y Tsan, Eileen M Stock, Jazmin M Gonzalez, David S Greenawalt, John E Zeber, Emran Rouf, Laurel A Copeland

**Affiliations:** 1 VISN 17 Center of Excellence for Research on Returning War Veterans, Department of Veterans Affairs, 4800 Memorial Drive (151C), Waco, TX 76711, USA; 2 VA Texas Valley Coastal Bend Health Care System, Department of Veterans Affairs, 2106 Treasure Hills Blvd., Harlingen, TX 78550, USA; 3 Central Texas Veterans Health Care System, Department of Veterans Affairs, 1901 Veterans Memorial Drive, Temple, TX 76504, USA; 4 Center for Applied Health Research, Scott & White Healthcare System, 2102 Birdcreek Drive Temple, TX 76502, USA; 5 Autism Comprehensive Educational Services, El Paso, TX 79912, USA; 6 Department of Internal Medicine, Scott & White Healthcare, 2401 S. 31st St., Temple, TX 76508, USA

**Keywords:** schizophrenia, veterans, quality of health care, mortality, primary care, preventive care

## Abstract

**Background:**

Schizophrenia is associated with excess mortality and multimorbidity, which is possibly associated with difficulty in coordinating care for multiple mental and physical comorbidities. We analyzed the receipt by patients with schizophrenia of 11 types of guideline-concordant care and the association of such care with survival.

**Methods:**

Guideline-concordant care over an 8-year period (financial years 2002 to 2009) was examined in a nationwide sample of 49,173 male veterans with schizophrenia, who were aged 50 years or older. Administrative databases from the electronic medical record system of the Veterans Health Administration (VA) provided comprehensive measures of patient demographics and medical information. Relying on the 2004 American Psychiatric Association guidelines, patterns in 11 types of care were identified and cluster-analyzed. Care types included cardiovascular, metabolic, weight management, nicotine dependence, infectious diseases, vision, and mental health counseling (individual, family, drugs/alcohol, psychiatric medication, and compensated work therapy). Survival analysis estimated association of care patterns with survival, adjusting for clinical and demographic covariates.

**Results:**

There was an average of four chronic diseases in addition to schizophrenia in the cohort, notably hypertension (43%) and dyslipidemia (29%). Three longitudinal trajectories (clusters) were identified: 'high-consistent' (averaging 5.4 types of care annually), 'moderate-consistent' (averaging 3.8), and 'poor-decreasing' (averaging 1.9). Most veterans were receiving cardiovascular care (67 to 76%), hepatic and renal function assays (79 to 84%), individual counseling (72 to 85%) and psychiatry consults (66 to 82%), with the proportion receiving care varying by cluster group. After adjustment for age, baseline comorbidity, and other covariates, there was a greater survival rate for those with poor-decreasing care compared with high-consistent care, and for high-consistent compared with moderate-consistent care.

**Conclusions:**

Relatively low levels of guideline-concordant care were seen for older VA patients with schizophrenia, and trajectories of care over time were associated with survival in a non-intuitive pattern. The group with the lowest and decreasing levels of care was also the oldest, but nonetheless had the best age-adjusted and other covariate-adjusted survival rates, possibly because they were requiring less care relative to younger, sicker veterans, and thus their comorbidity burden was markedly lower. Notably, in the group with the sickest individuals (that is those with the highest comorbidity scores, who were very disabled), receiving guideline-concordant care was associated with improved survival in adjusted models compared with those patients receiving only moderate levels of care.

## Background

Premature mortality among individuals with schizophrenia is well documented in the literature [1,2]. The increased mortality rates of these individuals is linked to their increased burden of medical [3,4] and psychiatric [5] comorbidities. Physical disorders in individuals with schizophrenia account for an estimated 60% of premature deaths [6], yet, these individuals are less likely to seek appropriate care and may use medical services intermittently, leading to unresolved medical problems, decreased treatment adherence, and premature mortality [7], which might be preventable with appropriate care.

To target the diverse health care needs of individuals with schizophrenia, the American Psychiatric Association (APA) outlined in 2004 practice guidelines for appropriate preventive care [8]. In addition to psychiatric medication management, the APA guidelines highlight the importance of comprehensive care for common conditions including hypertension [9,10], diabetes [11], obesity [12], lipid disorders [6], pulmonary and other infections [13], cardiovascular disease and metabolic syndrome [14], tobacco and substance use disorders [15], and liver disease [16]. Whether and which of these types of care are typically accessed and how they are related to survival remains to be studied.

Previous research on older patients with schizophrenia receiving care from the Veterans Health Administration (VA) noted associations of attendance at primary-care clinics with improved odds of survival, results that led to a directive for outreach to patients with schizophrenia who fail to access care for 12 months or more [3,17,18]. This study examined veterans with schizophrenia age 50 or older, receiving care in the VA, to determine whether there were particular types and patterns of routine outpatient care that were associated with improved survival, using a framework of specific care recommendations, over the 8-year period from October 2001 through September 2009.

## Methods

### Study design

The study was approved by the local institutional review board (IRB) before initiation. As part of a larger retrospective study of late-life healthcare for veterans with chronic disease [17], veterans diagnosed with schizophrenia and receiving VA care during the fiscal year (FY) 2002 (FY02; from October 2001 to September 2002) were followed to the end of FY09 using administrative extracts from the all-electronic medical records system of the VA.

### Study sample

Veterans seeking care from the VA were included in this study if they were diagnosed with schizophrenia (*International Classification of Disease *(ICD)9 code 295, excluding 295.5) on at least two outpatient or one inpatient dates during FY02, and were at least 50 years old in that year [19]. Exclusions were: death during FY02 (n = 2,051), female gender (n = 1,773), inconsistent dates with respect to death (probable data-entry errors; n = 134), missing data on veteran status (n = 125), and long-term inpatient status in FY02 (150+ days; n = 24) [20], leaving 49,173 patients (mean ± SD age 59.6 ± 9.2 years, range 50 to 102) in the cohort.

### Measures

Annual care indicators corresponded to APA categories [8]. Care type was captured from VA outpatient clinic identifiers (stop codes) and care provision codes (Current Procedural Terminology (CPT) and Healthcare Common Procedure Coding System (HCPCS); see Additional file 1). Recommendations for medical care addressed: weight management, tobacco cessation, infectious diseases (HIV, hepatitis C, or syphilis), cardiovascular risk (diabetes, hypertension, dyslipidemia, heart failure), and blood chemistries (renal and liver function, hyperprolactinemia). Eye care was also included, as older people require regular eye examinations, especially if they have co-occurring diabetes. Recommended mental health care included: individual psychotherapy, group counseling, family interventions, psychiatry medication consults, and drug/alcohol care or screening. The APA guidelines discuss the value of individual, group, and family counseling for patients with schizophrenia, emphasizing that treatment delivery must be tailored to the patient, leading to our selection of these five types of mental health care. Receipt of any care during a year constituted the meeting guidelines for that care type. The indicators were then summed to assess total annual care (range 0 to 11 for each year).

Given that antipsychotic treatment is strongly recommended for patients with schizophrenia, and medication adherence correlates with better outcomes [21], we also generated mutually exclusive covariates assessing baseline antipsychotic use: 1) good adherence was indicated by having the equivalent of 30-day prescriptions of antipsychotics (AP) for at least 10 months annually (10/12 or 83% adherence); 2) poor adherence was indicated by prescriptions for 1 to 9 months; and 3) irregular antipsychotic adherence. Irregular adherence was indicated by 0 months of AP use during the baseline year. Good adherence served as the reference group for the two indicators of suboptimal antipsychotic use. Gaps of 3 or more months in antipsychotic use (≤75% adherence) are associated with psychiatric readmission [21].

The primary outcome of interest (death) was based on the date of death as given in the VA Mini-Vital Status file, which is compiled from four sources using a validated algorithm for discordant cases. Sohn and colleagues [22] reported ~98% sensitivity for the VA file relative to the National Death Index. Survival was assessed in days.

We also assessed age, race, ethnicity, marital status, and VA priority score at baseline (FY02). VA priority, based on disability from a condition attributed to military service, describes why a veteran qualifies for VA care, and whether they have co-payments. It is a proxy for both socioeconomic status and disease severity [23]. Priority 1 veterans have a high level of disability, are often impoverished, and incur no co-payments for care or prescription medications in the VA. We contrasted priority 1 against all other priorities for VA care.

The case mix was assessed using the Charlson [24,25] and Selim [26] comorbidity indices. The Charlson sums weights for 19 conditions that are correlated with post-discharge mortality (including myocardial infarct, dementia, diabetes, and diabetes with complications) as implemented in administrative data [27,28]. The Selim sums 30 chronic physical and 6 psychiatric disorders as the Selim Comorbidity Score; this measure has been validated against care utilization, costs of care, and mortality [17]. The Charlson and Selim indices do not have high correlation with each other, and capture different aspects of the patient comorbidity burden.

### Analysis

Frequencies and means (standard deviations) were used to describe the baseline characteristics and types of care received each year. A cluster analysis uncovered longitudinal patterns in the level and trajectory of the 11 treatment types. For the cluster analysis, patterns among patients with complete data over the study period (survivors) were assessed, resulting in three clusters with eigenvalues of greater than 1 to denote significantly distinct patterns of care [29]. Patients with censored data were then examined in seven additional cluster analyses (one for each year after baseline) using data from all years before the year of death. For each year, the censored patterns were compared with the survivor patterns. Decedents were assigned to the survivor care cluster most similar to their pattern before death occurred; all decedent clusters had eigenvalues > 1 (graphs of the decedent trajectories are available upon request).

We examined Kaplan-Meier estimates of the time to death or to the end of the study (data right-censored) by care cluster. Both log-rank and Wilcoxon statistics showed significant differences between the three clusters (*P*<0.01). A Cox proportional-hazards regression model estimated risk-adjusted survival as a function of care cluster; covariates included age, Charlson and Selim comorbidity indices, medication adherence indicators, and VA priority 1 status.

## Results

### Sample and care descriptive

The Charlson and Selim comorbidity scores were 1.00 (SD ± ± 1.5 and 3.82 (SD ± 2.2, respectively, with an estimated correlation of *r *= 0.59 (35% shared variance; p<0.05). Slightly more than> half (53%) had VA priority 1 status (see Table 1).

**Table 1:** Baseline characteristics of veterans with schizophrenia aged 50 years or older (n = 49,173).

Parameter	
Age, years^1^	59.6 ± 9.2 (56.0) (50 to 102)
SCI^1^	3.82 ± 2.2 (3.0) (1 to 16)
CCS^1^	1.00 ± 1.5 (1.0) (0 to 16)

Ethnicity, n (%)	
White	32,245 (68.6)
Black	14,117 (30.0)
Hispanic	4,591 (9.3)
Other	658 (1.4)
Missing data	2,153
Marital status, n (%)	
Single (never married)	19,250 (39.8)
Married	13,333 (27.5)
Divorced	13,863 (28.6)
Widowed	1,960 (4.0)
Missing data	767
Priority 1 status	26,187 (53.3)
Antipsychotic adherence, n (%)	
Good	13,086 (26.6)
Poor	27,349 (55.6)
Irregular	8,738 (17.8)
Medical/physical disorders, n (%)	
Anemia	2,935 (6.0)
Angina	1,148 (2.3)
Cancer	3,169 (6.4)
Cataracts	5,279 (10.7)
Congestive heart failure	1,595 (3.2)
COPD	7,482 (15.2)
Diabetes mellitus (per SCI)	11,423 (23.2)
Hepatitis	2,786 (5.7)
Hypertension	21,062 (42.8)
Dyslipidemia	14,147 (28.8)
Cardiac arrhythmias	1,940 (3.9)
Lower back pain	5,872 (11.9)
Myocardial infarction	1,147 (2.3)
Nicotine dependence	11,429 (23.2)
Obesity	5,920 (12.0)
Peripheral vascular disease	1,399 (2.8)
Chronic renal disease	1,344 (2.7)
Stroke	1,422 (2.9)
Urinary tract infection	1,484 (3.0)
Mental health disorders	
Alcohol abuse or dependence	6,448 (13.1)
Anxiety	4,221 (8.6)
Bipolar disorder	5,998 (12.2)
Depression	10,488 (21.3)
PTSD	6,075 (12.4)

Abbreviations: CCS, Charlson Comorbidity Score; COPD, chronic obstructive pulmonary disease; SCI, Selim Comorbidity Index; PTSD, post-traumatic stress disorder.

^1^Data are mean ± SD (median) (range).

**Figure 1:**
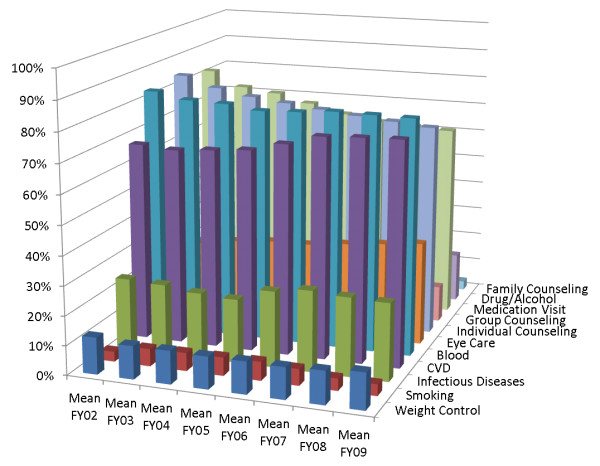
**Proportion of patients with schizophrenia receiving each type of care, stratified by fiscal year (n = 49,173)**.

In terms of guideline-concordant care for FY02 to FY09, most of the veterans were receiving cardiovascular care (67 to 76%), hepatic and renal function assays (79 to 84%), individual counseling (72 to 85%), and psychiatry consults (66 to 82%). Family counseling, individual counseling, group counseling, and psychiatry consults appeared to decrease over time (from 82% with psychiatric consults in FY02 to 66% in FY09), whereas cardiovascular and drug/alcohol care appeared to increase (see Figure 1). Only a small proportion of VA patients in this study had documented care for nicotine dependence (3 to 6%), weight management (11 to 13%), drug/alcohol use (13 to 17%), infectious disease (22 to 28%), or eye care (26 to 35%), or received group (12 to 21%) or family counseling (3 to 8%),.

### Cluster profiles

**Figure 2:**
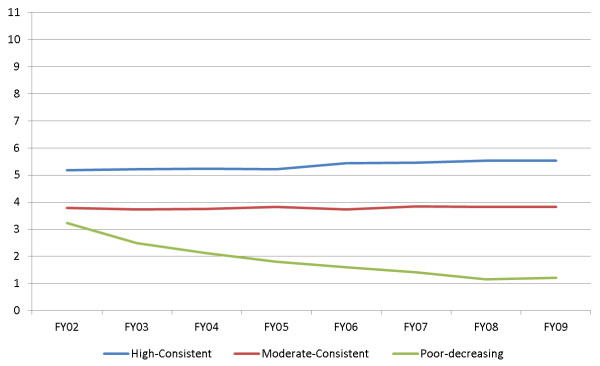
**Cluster trajectories of patients with schizophrenia receiving care, stratified by fiscal year (n = 49,173)**.

Individuals with high-consistent care averaged 5.4 types of care across all years (Figure 2). This was the most common pattern, evident in the youngest group with the most comorbidity and the largest proportion of priority 1 veterans (n = 20,854). This group experienced 26% mortality over the follow-up period (unadjusted rate). Those with moderate-consistent care averaged 3.8 types of care, and these were a group of veterans with an unadjusted mortality rate of 36% (n = 18,218). People in the poor-decreasing trajectory averaged 1.9 types of care across all years; this group had the oldest patients (but with a wider variation in age, with SD = 10 years), but had the least comorbidity and smallest proportion of priority 1 veterans, and experienced an unadjusted mortality rate of 31% (n = 10,101). Cancer was relatively more common in the moderate-consistent care group whereas cardiovascular diagnoses were highest in the high-consistent care group (Table 2).

**Table 2:** Baseline patient characteristics by clusters (n = 49,173).

	high-consistent care, n = 20,854	Moderate-consistent care, n = 18,218	poor-decreasing care, n = 10,101
Age, years^1^	57.8 ± 8.0	60.3 ± 9.5	62.2 ± 10.1
SCI^1^	4.37 ± 2.3	3.48 ± 2.1	3.31 ± 2.1
CCS^1^	1.16 ± 1.6	0.91 ± 1.5	0.82 ± 1.3

Ethnicity, n (%)			
White	13,602 (65.2)	12,069 (66.3)	6,574 (65.1)
Black	6,529 (31.3)	5,000 (27.5)	2,588 (25.6)
Other	289 (1.4)	242 (1.3)	127 (1.3)
Hispanic	2,518 (12.1)	1,513 (8.3)	560 (5.5)
Unknown	434 (2.1)	907 (5.0)	812 (8.0)
Marital status			
Single (never married)	7,548 (36.2)	7,243 (39.8)	4,459 (44.1)
Married	6,317 (30.3)	4,937 (27.1)	2,079 (20.6)
Divorced	6,049 (29.0)	4,945 (27.1)	2,869 (28.4)
Widowed	703 (3.4)	765 (4.2)	492 (4.9)
Priority 1 status	12,232 (58.7)	9,616 (52.8)	4,339 (43.0)
Mortality	5,491 (26.3)	6,585 (36.2)	3,126 (31.0)
Antipsychotic adherence			
Good	6,640 (31.8)	4,737 (26.0)	1,709 (16.9)
Poor	11,700 (56.1)	10,203 (56.0)	5,446 (53.9)
Irregular	2,514 (12.1)	3,278 (18.0)	2,946 (29.2)
Medical/physical diagnoses			
Anemia	1,348 (6.5)	1,071 (5.9)	516 (5.1)
Angina	648 (3.1)	348 (1.9)	152 (1.5)
Cancer	1,481 (7.1)	1,237 (6.8)	451 (4.5)
Cataracts	2,932 (14.1)	1,555 (8.5)	792 (7.8)
Congestive heart failure	715 (3.4)	613 (3.4)	267 (2.6)
COPD	3,526 (16.9)	2,753 (15.1)	1,203 (11.9)
Diabetes mellitus per Selim	6,724 (32.2)	3,217 (17.7)	1,482 (14.7)
Hepatitis	1,676 (8.0)	796 (4.4)	314 (3.1)
Hypertension	10,876 (52.2)	7,128 (39.1)	3,058 (30.3)
Dyslipidemia	7,999 (38.4)	4,547 (25.0)	1,601 (15.9)
Cardiac arrhythmias	962 (4.6)	660 (3.6)	318 (3.2)
Lower back pain	3,269 (15.7)	1,835 (10.1)	768 (7.6)
Myocardial infarction	528 (2.5)	431 (2.4)	188 (1.9)
Nicotine dependence	5,897 (28.3)	3,770 (20.7)	1,762 (17.4)
Obesity	3,614 (17.3)	1,649 (9.1)	657 (6.5)
Peripheral vascular disease	658 (3.2)	485 (2.7)	256 (2.5)
Chronic renal disease	616 (3.0)	503 (2.8)	225 (2.2)
Stroke	596 (2.9)	488 (2.7)	338 (3.4)
Urinary tract infection	662 (3.2)	548 (3.0)	274 (2.7)
Mental health disorders			
Alcohol	3,507 (16.8)	1,859 (10.2)	1,082 (10.7)
Anxiety	2,210 (10.6)	1,357 (7.5)	654 (6.5)
Bipolar disorder	3,163 (15.2)	1,796 (9.9)	1,039 (10.3)
Depression	5,548 (26.6)	3,199 (17.6)	1,741 (17.2)
PTSD	3,541 (17.0)	1,744 (9.6)	790 (7.8)

Abbreviations: CCS, Charlson Comorbidity Score; COPD, chronic obstructive pulmonary disease; SCI, Selim Comorbidity Index; PTSD, post-traumatic stress disorder.

^1^Data are mean ± SD (median) (range).

Patients in the high-consistent group consistently received more types of care over time, including weight management (18% to 21% per year), infectious disease (32% to 41%), renal/liver function (95% to 99%), and individual counseling (93 94%), compared with those in the moderate-consistent (6% to 8%, 18% to 22%, 80% to 88%, and 74% to 82%, respectively) or poor-decreasing groups (2% to 8%, 6% to 16%, 28% to 65%, and 13% to 72%, respectively). Overall, there was an apparent increasing trend from baseline to FY09 in the proportion of patients receiving cardiovascular care (from 83% to 96%), eye care (37% to 55%), drug/alcohol care (20% to 27%) and renal/liver function care (95% to 99%). However, a decreasing trend was seen for psychiatric medication consults (91% to 88%), group counseling (31% to 23%), and family counseling (11% to 5%). Weight management, nicotine dependence, and individual counseling appeared fairly stable across the years. A smaller proportion of patients in the poor-decreasing group received recommended treatment across all types of care than in the high-consistent and moderate-consistent groups. In the poor-decreasing group, a moderate percentage of patients initially received several types of recommended care, but this proportion dropped sharply over time from baseline to FY09: cardiovascular care dropped from 48% to 24%, renal/liver function care from 65% to 29%, psychiatric medication management from 70% to 20%, and individual counseling from 72% to 24%. In the poor-decreasing group, the numbers receiving weight-management care (8% to 2%) and eye care (16% to 7%) were particularly low.

### Mortality models

The relationship between receipt of guideline-concordant care and mortality rate was examined with and without adjustment for baseline characteristics, using Cox proportional-hazards models. In the unadjusted model, the estimated probability of surviving at least 5 years was 0.85 for those in the high-consistent group, 0.77 in the moderate-consistent groups, and 0.82 in the poor-decreasing group. Survival from Oct 2001 until death averaged 7.1 years for the high-consistent, 6.6 years for the moderate-consistent, and 6.9 years for the poor-decreasing group. The 25th percentile of survival time was estimated at 7.6 years for the high-consistent group, 5.5 years for the moderate-consistent group, and 6.3 years for the poor-decreasing group.

**Figure 3:**
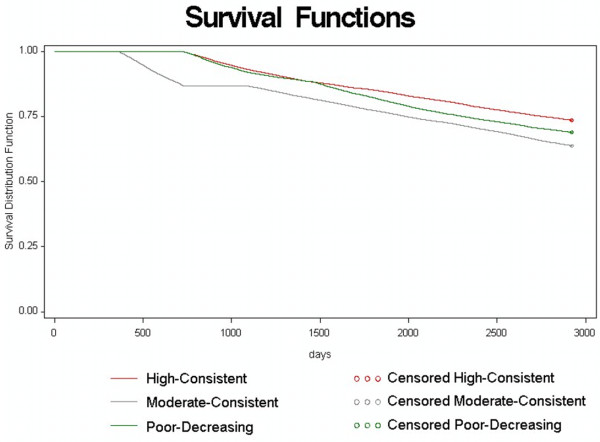
**Survival function of the three patterns of care (n = 49,173)**.

In the multivariable adjusted model, the hazard ratio (HR) for the moderate-consistent group indicated a 41% increase in the mortality rate (HR = 1.41, 95% CI 1.36 to 1.47) relative to the high-consistent group (see Table 3). However, patients in the poor-decreasing group experienced a 6% decrease in mortality rate (HR = 0.94; 95% CI 0.90 to 0.99) relative to the reference (high-consistent) group,. These results varied from those of the unadjusted survival analysis. Whereas the unadjusted survival findings showed that patients receiving consistently frequent care had the greatest survival rate (Figure 3), the adjusted model showed that patients in the poor-decreasing care group had a lower mortality rate. Mortality also correlated with age (increasing by 78% per decade) and comorbid disease burden. For each unit increase in the Charlson and Selim scores, mortality increased by 21% and 1%, respectively.

**Table 3:** Cox proportional-hazards model for mortality (*n *= 49,173).

Predictor	Estimate, mean ± SE	HR (95% CI)	*P *value
Age (in decades)	0.58 (0.01)	1.78 (1.75 to 1.81)	<0.01
CCS	0.19 (<0.01)	1.21 (1.20 to 1.23)	<0.01
SCI total	0.01 (<0.01)	1.01 (1.00 to 1.02)	<0.01
Priority 1 status	-0.09 (0.02)	0.91 (0.89 to 0.94)	<0.01
AP adherence			
Poor	0.08 (0.02)	1.08 (1.04 to 1.12)	<0.01
Irregular	0.15 (0.02)	1.16 (1.10 to 1.21)	<0.01
Type of care			
Moderate-consistent	0.35 (0.02)	1.41 (1.36 to 1.47)	<0.01
Poor-decreasing	-0.06 (0.02)	0.94 (0.90 to 0.99)	0.02

Abbreviations: AP, Antipsychotic; CCS, Charlson Comorbidity Score; HR, hazard ratio; SCI, Selim Comorbidity Index.

## Discussion

For older male outpatients with schizophrenia, our analyses support a modest longitudinal association over an 8-year period between a greater scope of guideline-concordant care and survival. However, patients in the group who received the least comprehensive guideline-concordant care had better adjusted odds of survival than those receiving high or moderate levels of care, although the benefit relative to those with high-consistent care was small. This group was characterized by lower comorbidity, older age (but a larger age range), and fewer VA priority 1 veterans (those with a high level of disability), thus apparently less need for care. The mechanisms by which this survival benefit might accrue include access to care being selectively used, emphasis on prevention and early disease management being common in primary care, and a focus on necessary care [30]. Possibly these patients also avoided excess medical care that might itself carry increased risk to their well-being through iatrogenic mechanisms. Moreover, patients in the poor-decreasing care group had less comorbidity, which is known to account for half the excess mortality of persons with schizophrenia [31]. Their relatively lower rates of cardiovascular diagnoses may account for some of the findings and the lower levels of care.

Interestingly, this sample of veterans with schizophrenia received on average only 4.8 out of the 11 care types examined. The patient's burden of illness translates into the healthcare system's challenge of providing appropriate preventive services; difficult care coordination for patients with the diminished self-care capacity or cognitive deficits common with schizophrenia may account for this modest level of guideline-concordant care [32]. Least common were visits for nicotine dependence, weight management, drug/alcohol dependency infectious diseases, eye care, and group psychotherapy. Over time, possibly due to aging, the percentage of patients receiving cardiovascular and eye care tended to increase, which is appropriate given the age-related nature of cardiovascular and eye disorders. The failure to address weight management is unfortunate, as this common condition potentiates much cardiovascular disease. Other research on VA patients with schizophrenia noted no disparities in receipt of obesity care practices, but did note a generally low level of weight management [33].

Of note, the proportion of patients receiving mental health treatment (psychiatric consults for medication management, and individual, family, and group therapy) gradually decreased over time. Regarding medication management, this may indicate diminishing use of antipsychotic agents in later life. A 2004 review suggested that older persons with schizophrenia do not need fewer medications, but a recent analysis suggests that they may require lower doses or antipsychotics or none at all [34,35]. Nevertheless, patients who used antipsychotics consistently had a longer survival than those with poor or inconsistent use, seemingly in line with the report by Maher and Theodore, who found that appropriate antipsychotic use was not associated with increased mortality [36]. The results from liver and renal function tests, often used to monitor adverse drug effects, were generally high [37]. Families involved in the care of these patients should be mindful of the need to work closely with care providers regarding medication options (for example, dose adjustment, possible medication changes to limit weight gain). Psychological counseling, particularly family counseling, can improve illness insight and thence treatment adherence, and also improve family functioning [38]. The declines in mental health counseling merit investigation into the client and family need for and benefit from these services.

Patients who fail to come in for outpatient care for 12 months or more [17] experience heightened risk of death, whether through increased risk due to heart disease [39], adverse effects of antipsychotics on cardiovascular functioning and metabolic regulation [40,41], or increased risk of liver or renal disease [16]. Over time, the proportion of patients receiving renal/liver function care and psychiatric medication management in the moderate-consistent and poor-decreasing care groups gradually declined. Receiving liver function screening, especially for patients with liver disease, is associated with appropriate treatment and longer survival [42]. Insufficient monitoring would provide fewer opportunities to make adjustments in medication regimens including hepatotoxic medications, and patients are likely to continue using psychotropic medications that exacerbate liver problems if poor liver functioning goes undetected. Accordingly, providers and family caretakers should be vigilant when patients decrease their healthcare visits over time or miss appointments.

As this is perhaps the first study to examine the association of comprehensive guideline-based care with survival for older male patients with schizophrenia, our findings suggest that greater adherence to screening and treatment recommendations is crucial to patient survival, and may reduce premature mortality. Additionally, early detection and treatment of incipient problems is likely to produce greater returns in terms of quality of life for older patients with schizophrenia, and to reduce the strain of caretaking for the older patient's social network. It is important for mental and primary healthcare practitioners to coordinate care efforts and improve overall adherence to these treatment guidelines. These patients may benefit from aggressive outreach and use of a patient-centered medical home to improve care coordination and treatment adherence.

### Limitations and strengths

This study relied on administrative data, thus severity ratings of psychiatric or medical illnesses were not available and nor were quality-of-life assessments. Medicare and other out-of-system data were unavailable, thus any such services received were not captured by our study methods [23]. Future research linking VA with Medicare data to assess guideline-concordant care could determine the extent of bias introduced in our results by reliance on a single healthcare system for those persons covered by Medicare. For example, patients in the poor-decreasing care cluster may have been more likely than others to seek healthcare services outside the VA after the baseline year, as many were reaching the age of 62 (early eligibility) or 65 (usual eligibility) years during the period of observation. On the other hand, patients with serious mental illness tend to stay within the VA [43]. Although a high proportion of patients received family counseling, it is uncertain whether this translated into evidenced-based family services for schizophrenia, considering that previous studies have identified low rates of such care [44]. Of note, the present study focused on male veterans age 50 years or older, therefore results cannot be generalized to women or younger patients. VA patients tend to have lower incomes and more health problems than the general population in the USA, which may affect their need for care and the breadth of healthcare options available to them [45].

The strengths of the study include its large scale and longitudinal design, which enabled us to follow patients over an 8-year period, and an innovative approach mapping the treatment recommendations from APA guidelines onto the indicators of care received. The study addressed a crucial and serious healthcare problem, namely, premature mortality among patients with schizophrenia. We were careful to include preventive care, such as screenings for common medical and psychiatric comorbidities, as well as treatment for these conditions. A further strength was the use of cluster analysis, which enabled a practical and interpretable comparison of patient groups characterized by different patterns of care over time.

Future research on the effects of preventive care for individuals with schizophrenia is needed to replicate and extend the results of this study. Studies are needed in other healthcare systems, and including people who lack access to consistent sources of care. Also of value would be an examination of the relative importance of the types of recommended care in reducing premature mortality. For example, through family counseling, family members of patients with schizophrenia may be more likely to assist with medical adherence and coordination. This may be especially important when patients with schizophrenia require complex coordinated care, which may be difficult to manage.

## Conclusions

Older veterans with schizophrenia received only a modest number of the recommended annual health care services in the VA. Patients receiving consistent and more comprehensive guideline-concordant care had better survival than those receiving moderate-consistent levels of care, even though they had more illnesses. These findings highlight the importance of the quality and comprehensiveness of guideline-concordant preventive healthcare for older patients with schizophrenia, augmenting the literature, which has previously shown that receipt of primary care correlates with survival [17,46,47]. Healthcare systems should consider ways to better engage patients and their families in coordinated healthcare services. Barriers to access, treatment needs, and treatment preferences must be addressed creatively to maintain the highest quality of care for our most vulnerable patients.

## Abbreviations

APA: American Psychiatric Association; CPT: Current Procedural Terminology; FY: Fiscal year; HCPCS: Healthcare Common Procedure Coding System; HIV: Human immunodeficiency virus; IRB: Institutional review board; VA: Veterans Health Administration.

## Competing interests

The authors on this study have no financial conflict of interests for the past 3 years. The views expressed in this article are those of the authors, and do not necessarily reflect the position or policy of the Department of veterans Affairs or the US government.

## Authors' contributions

The idea was conceived by JT, JG, JZ, and LC. The literature search was conducted by JT, JG, JZ, and DG. ES and LC provided conceptual feedback, analysis, and design. JT, ES, LC, JG, DG, and ER reviewed and approved all CPT, HCPCS, and Stop codes. All authors read and approved the final version of the manuscript.

## Authors' information

JT was affiliated with the VISN 17 Center of Excellence for Research on Returning War veterans, Department of veterans Affairs in Waco, TX at the time of the study. JT is now at VA Texas Valley Coastal Bend Health Care System at the Harlingen Outpatient Primary Care Clinic.

## Pre-publication history

The pre-publication history for this paper can be accessed here:

http://www.biomedcentral.com/1741-7015/10/147/prepub

## Supplementary Material

Additional file 1 **Veterans Health Administration (VA) stop and Current Procedural Terminology (CPT) codes**. Type of care and associated VA stop codes or CPT codes Click here for file
