# Supplementary material for: Mortality and guideline-concordant care for older patients with schizophrenia: a retrospective longitudinal study
Source: BMC Med. 2012 Nov 26;10:147. doi: 10.1186/1741-7015-10-147 (PMC3523058; doi:10.1186/1741-7015-10-147)
Supplement: Additional file 1 — Veterans Health Administration (VA) stop and Current Procedural Terminology (CPT) codes. Type of care and associated VA stop codes or CPT codes [file 1741-7015-10-147-S1.DOC]

**Additional File 1: VA stop and CPT codes**

**Type of care and associated VA stop codes or CPT codes**

| **MEDICAL HEALTH CARE** | | |
| --- | --- | --- |
| **Type of Care** | **VA Stop Code** | **CPT/HCPCS Code** |
| Weight Management/Control | 123: nutr/diet - individual  124: nutr/diet – group  139: weight control  140: phys.fit/exer.  175: HBPC Dietician | 97802: Med Nutr Ther Initial Assmt/Intervention  97803: Med Nutr Ther ReAssess/Intervention  97804: Med Nutr Ther Group  S9449: (HCPCS Code) Weight Mgmt Class  S9452: Nutrition Class  S9451: Exercise Class  S9470: Nutritional Counseling, Diet  G0270: individual medical nutrition therapy  G0271: Medical nutrition therapy, reassessment and subsequent intervention  2001F: WEIGHT RECORD  D1310: NUTRITIONAL COUNSELING FOR T  G0112: Nett;nutrition guid, initial  G0113: Nett;nutrition guid,subseqnt |
| Nicotine Dependence | 138: smoking cessation  533: smoking cessation ind  707: smoking cessation | 1000F: Tobacco use assessed  1034F: Smoking Status and Cessation Advice or Treatment indicator  4000F: Tobacco use cessation intervention, counseling  4001F: Tobacco use cessation intervention, pharmacologic therapy  4004F: Tobacco cessation counseling  G0375: (HCPCS Code) Tobacco cessation counseling  G0376: (HCPCS Code) Tobacco cessation counseling  99406: Smoking and tobacco-use cessation counseling visit  99407: Smoking and tobacco-use cessation counseling visit  G0436: Counseling for tobacco  G0437: counseling for tobacco  G9016: (HCPCS Code) Smoking cessation counseling, individual, in the absence of or in addition to any other evaluation and management service, per session  S4995: (HCPCS Code) SMOKING CESSATION GUM  S4991: (HCPCS Code) Nicotine patch nonlegend  S4990: (HCPCS Code) NICOTINE PATCH LEGEND  D1320: TOBACCO COUNSELING  S9075: (HCPCS Code) SMOKING CESSATION TREATMENT  S9453: (HCPCS Code) SMOKING CESSATION CLASS |
| Infectious Diseases   - HIV - Hepatitis C - Syphilis | 112: inf dis, nuc med  310: infectious dis | HIV  0575F: HIV RNA control plan of care, documented (HIV)  3492F: HIV screening and cell counts. History of nadir CD4+ cell count <350 cells/mm (HIV)  3493F: HIV screening and cell counts. No history of nadir CD4+ cell count <350 cells/mm AND no history of AIDS-defining condition (HIV)  3494F: HIV screening and cell counts. CD4+ cell count <200 cells/mm (HIV).  3495F:  86689: Antibody; HTLV or HIV antibody, confirmatory test  86701: Antibody; HIV-1  86702: Antibody; HIV-2  86703: Antibody; HIV-1 and HIV-2, single assay  87390: Infectious agent antigen detection by enzyme immunoassay technique, qualitative or semiquantitative, multiple-step method; HIV-1  87391: Infectious agent antigen detection by enzyme immunoassay technique, qualitative or semiquantitative, multiple-step method; HIV-  87534: Infectious agent detection by nucleic acid (DNA or RNA); HIV-1  87535: Infectious agent detection by nucleic acid (DNA or RNA); HIV-1  87536: Infectious agent detection by nucleic acid (DNA or RNA); HIV-1  87537: Infectious agent detection by nucleic acid (DNA or RNA); HIV-2  87538: Infectious agent detection by nucleic acid (DNA or RNA); HIV-2  87539: Infectious agent detection by nucleic acid (DNA or RNA); HIV-2  87901: Infectious agent genotype analysis by nucleic acid (DNA or RNA); HIV-1, reverse transcriptase and protease regions  87903: Infectious agent phenotype analysis by nucleic acid (DNA or RNA) with drug resistance tissue culture analysis, HIV 1;  87904: Infectious agent phenotype analysis by nucleic acid (DNA or RNA) with drug resistance tissue culture analysis, HIV 1  87906: Infectious agent genotype analysis by nucleic acid (DNA or RNA); HIV-1, other region (eg, integrase, fusion)  88363: Examination and selection of retrieved archival (ie, previously diagnosed) tissue(s) for molecular analysis (eg, KRAS mutational analysis)  G0435: HIV screening  G0432: HIV screening  G0433: HIV screening |
|  |  | AIDS  G8491: Intend To Report The Hiv/Aids Measures Group  G8500: All quality actions for the applicable measures in the hiv/aids measures group have been performed for this patient  3490F: History of AIDS-defining condition |
|  |  | Hepatitis C  3218F: RNA testing for Hepatitis C documented as performed within 6 months prior to initiation of antiviral treatment for Hepatitis C (HEP-C)  3220F: Hepatitis C quantitative RNA testing documented as performed at 12 weeks from initiation of antiviral treatment (HEP-C)  3265F: Ribonucleic acid (RNA) testing for Hepatitis C viremia ordered or results documented (HEP C)  3266F: Hepatitis C genotype testing documented as performed prior to initiation of antiviral treatment for Hepatitis C (HEP C)  3514F: Hepatitis C screening documented as performed (HIV)  3515F: Patient has documented immunity to Hepatitis C (HIV)  4150F: Patient receiving antiviral treatment for Hepatitis C (HEP-C)  80074: Acute hepatitis panel  86803: Hepatitis C antibody  86804: Hepatitis C confirmatory test  87520: Infectious agent detection by nucleic acid (DNA or RNA); hepatitis C, direct probe technique  87521: Infectious agent detection by nucleic acid (DNA or RNA); hepatitis C, amplified probe technique  87522: Infectious agent detection by nucleic acid (DNA or RNA); hepatitis C, quantification  87902: Infectious agent genotype analysis by nucleic acid (DNA or RNA); Hepatitis C virus  80076: Hepatic Function Panel  G8459: Clinician Documented That patient is receiving antiviral treatment for hepatitis C  G8461: Patient receiving antiviral treatment for hepatitis C  G8462: Clinician documented that patient is not an eligible candidate for counseling regarding contraception prior to antiviral treatment; patient not receiving antiviral treatment for hepatitis C  G8463: Patient receiving antiviral treatment for hepatitis C documented  G8545: Intend to report the hepatitis C measures group  G8549: All quality actions for the applicable measures in the hepatitis C measures group have been performed for this patient  86303: Hepatitis C Antibody |
|  |  | Syphilis  3512F: Syphilis screening documented as performed (HIV)  80055: Syphilis test  86592: Syphilis test  86593: Syphilis test |
| Care for Cardiovascular Risk Factors   - Diabetes - Hypertension - Cholesterol - Heart failure | Diabetes  305: endocr/metab  306: diabetes  Hypertension  129: hypertension scr  309: hypertension  701: hyperten screen/bp ck  Heart  107: EKG  110: Card/vas nuc med  303: cardiology  334: cardiac stress test  421: vascular lab | Diabetes  83036 - Glycosylated Hemoglobin Test  83037 - Hemoglobin; glycosylated (A1C) by device cleared by FDA for home use  3044F - Most recent hemoglobin A1c (HbA1c) level less than 7.0% (DM)  3045F – Hg a1c level 7.0-9.0%  3046F - Most recent hemoglobin A1c level greater than 9.0% (DM)  5010F - Macul result to phy mng dm  G0108 - Diab Manage Trn Per Indiv  G0109 - Diab Manage Trn Ind/Group  G8485 - Report, Diabetes measures  G8494 - DM meas qual act perform  S0390 - Routine Foot Care Per Visit  95250 - Glucose monitoring  S9455 - Diabetic management program  S9460 - Diabetic management program/nurse  S9465 - Diabetic management program/dietician  S9140 Diabetic Management Program  S9141 Diabetic Management Program  82947 - Assay, Glucose, Blood Quant |
|  |  | LDL Screen  80061 - Lipid Panel  83700 - Lipopro bld electrophoretic  83701 - Lipoprotein bld hr fraction  83704 - Lipoprotein bld by nmr  83721 - Assay of blood lipoprotein  3048F - Ldl-c <100 mg/dl  3049F - Ldl-c 100-129 mg/dl  3050F - Ldl-c >= 130 mg/dl  S2120 Low Density Lipoprotein (LDL) |
|  |  | Cardiovascular  78499 - Cardiovascular Nuclear Exam  93015 - Cardiovascular Stress Test  93016 - Cardiovascular Stress Test  93017 - Cardiovascular Stress Test  93018 - Cardiovascular Stress Test  93350 - Echo Transthoracic  93351 - Echocardiography, transthoracic, real-time with image documentation (2D), includes M-mode recording, when performed, during rest and cardiovascular stress test using treadmill  93660 - Evaluation of cardiovascular function with tilt table evaluation, with continuous ECG monitoring and intermittent blood pressure monitoring, with or without pharmacological intervention  93701 - Bioimpedance, Thoracic  93799 - Unlisted Cardiovascular Service Or Procedure  3120f - 12-Lead ECG Performed  93000 - Electrocardiogram, Complete  93005 - Electrocardiogram, Tracing  93040 - Rhythm ECG With Report  93041 - Rhythm ECG, Tracing  93797 - Cardiac Rehab  93798 - Cardiac Rehab/Monitor  93010 Electrocardiogram Report  93012 Transmission Of ECG  93014 Report On Transmitted ECG  93024 Cardiac Drug Stress Test  93561 Cardiac Output Measurement  93562 Cardiac Output Measurement  93571 Heart Flow Reserve Measure  93572 Heart Flow Reserve Measure  S9472 Cardiac Rehabilitation Progr  75556 Cardiac MRI/Flow Mapping  75554 Cardiac MRI/Function  78459 Heart Muscle Imaging (Pet)  78464 Heart Image (3d), Single  78465 Heart Image (3d), Multiple  78492 Heart Image (PET), Multiple  78494 Heart Image, Spect  93307 Echo Exam Of Heart  93308 Echo Exam Of Heart  93320 Doppler Echo Exam, Heart  93321 Doppler Echo Exam, Heart |
|  |  | Hypertension  4050F - Hypertension plan of care documented as appropriate (HTN) |
|  |  | Cholesterol  3011F - Lipid panel doc rev  82465 - Assay, Bld/Serum Cholesterol  83718 - Assay Of Lipoprotein  83719 - Assay Of Blood Lipoprotein  83721 - Assay Of Blood Lipoprotein  G8593 - Lipid pn results |
|  |  | Heart failure  0001F - Heart failure composite  4003F - Patient education, written/oral, appropriate for patients with heart failure, performed (HF)  4014F - Written discharge instructions provided to heart failure patients discharged home (Instructions include all of the following components: activity level, diet, discharge medications, follow-up appointment, weight monitoring, what to do if symptoms worsen)  G8548 - HF measures grp  G8551 - HF MG qual act perform  S9109 - CHF Telemonitoring Month |
| Blood Chemistries   - Renal function - Liver function - Hyperprolactinemia | 108: laboratory  308: hematology | 78220 - Liver Function Study  80069 - Renal Function Panel  82040 - Assay Of Serum Albumin  82565 - Assay Of Creatinine |
|  |  | Hyperprolactinemia  80440 - Thyrotropin releasing hormone (TRH) stimulation panel; for hyperprolactinemia This panel must include the following: Prolactin (84146 x 3) |
| Eye Care | 408: optometry  437: visual impairment ctr to optimize remaining sight  439: low vision care  718: diabetic retinal screening | 92002 - Eye Exam, New Patient  92004 - Eye Exam, New Patient  92012 - Eye Exam  92014 - Eye Exam & Treatment  92018 - New Eye Exam & Treatment  92019 - Eye Exam & Treatment  92020 - Special Eye Evaluation  92225 - Special Eye Exam, Initial  92226 - Special Eye Exam, Subsequent  92230 - Eye Exam With Photos  92235 - Eye Exam With Photos  92240 - Icg Angiography  92250 - Eye Exam With Photos  92260 - Ophthalmoscopy/Dynamometry  S0620, S0621 - Routine ophthalmological exam  S0625 - Digital screening retina  S3000 - Bilat dil retinal exam  2022F - Dil retina exam interp rev  2024F - 7 field photo interp doc rev  2026F - Eye image valid to dx rev  3072F - Low risk for retinopathy  4174F - Couns potent glauc impct  4176F - Counseling about value of protection from UV light and lack of proven efficacy of nutritional supplements in prevention or progression of cataract development provided to patient and/or caregiver(s)  4177F - Counseling about the benefits and/or risks of the Age-Related Eye Disease Study (AREDS) formulation for preventing progression of age-related macular degeneration (AMD) provided to patient and/or caregiver(s) |

| **MENTAL HEALTH CARE** | | |
| --- | --- | --- |
| **Type of Care** | **VA Stop Code** | **CPT Code** |
| Individual Counseling   - Individual counseling - Compensated work therapy (CWT) | 510: psychology ind  502: mental health ind  505: day trmt indi  520: lt enhance indiv  531: mh prim care team ind  532: psyc/soc rehab-ind  576: psychogeria cli/indv  CWT  515: CWT/TR-HCMI  568: MH CWT/supported employment (CWT/SE) face-to-face  569: MH CWT/SE non face-to-face CBO non-count  570: MH CWT/transitional work experience non face-to-face CBO non-count  574: MH CWT /transitional work experience, face-to-face | 90804: Psychiatric Treatment, Office, 20-30 MIN  90805: Psychotherapy, Office, 20-30 MIN W/E  90806: Psychotherapy, Office, 45-50 MIN  90807: Psychotherapy, Office, 45-50 MIN W/E  90808: Psychotherapy, Office, 75-80 MIN  90809: Psychotherapy, Office, 75-80, W/E  90810: Interactive Psychotherapy, OFF, 20-30 MIN  90811: Interactive Psychotherapy, 20-30, W/E  90812: Interactive Psychotherapy, OFF, 45-50 MIN  90813: Interactive Psychotherapy, 45-50 MIN W/E  90814: Interactive Psychotherapy, OFF, 75-80 MIN  90815: Interactive Psychotherapy, 75-80 W/E  90816: Psychotherapy, HOSP, 20-30 MIN  90817: Psychotherapy, HOSP, 20-30 MIN W/E  90818: Psychotherapy, HOSP, 45-50 MIN  90819: Psychotherapy, HOSP, 45-50 MIN W/E  90820: Diagnostic Interview  90821: Psychotherapy, HOSP, 75-80 MIN  90822: Psychotherapy, HOSP, 75-80 MIN W/E  90823: Interactive Psychotherapy, HOSP, 20-30 MIN  90824: Interactive Psychotherapy, HOSP 20-30 W/E  90826: Interactive Psychotherapy, HOSP, 45-50 MIN  90827: Interactive Psychotherapy, HSP 45-50 W/E  90828: Interactive Psychotherapy, HOSP, 75-80 MIN  90829: Interactive Psychotherapy, HOSP, 75-80 W/E  96100: Psychological Testing  96101: Psychological Testing  G0177: OPPS/PHP; train & educ serv (day treatment)  96150: Health and behavior assessment (e.g., health-focused clinical interview, behavioral observations, psychophysicological monitoring, health-oriented questionnaires), each 15 minutes, face-to-face with the patient |
| Group counseling | 516: PTSD group  521: LT enhance group  553: Day TRMT-grp  554: Day Hospital-grp  558: psychology-grp  559: ps/soc rehab-grp  561: PCT PTSD-grp  563: MH prim care team-grp  539: MH Integrated Care- Group  577: Psychogeriatric Clinic- Group | 90853: Group psychotherapy (other than of a multiple-family group)  90857: Interactive group psychotherapy  G0410: Grp psych partial hosp 45-50  G0411: Inter active grp psych parti  H0046: Mental Health Service, NOS (Group Community Support) |
| Family Counseling |  | 90847: Family Psychotherapy W/Patient  90849: Multiple Family Group Psychotherapy  T1006: Family/Couple Counseling  T1027: Family training & counseling  90887: Interpretation or explanation of results of psychiatric, other medical examinations and procedures, or other accumulated data to family or other responsible persons, or advising them how to assist patient  90846: Family Psychotherapy W/O Patient  96154: Interv Hlth/Behav, Fam W/Pt  96155: Interv Hlth/Behav Fam No Pt  H1011 Family assessment  S5110 Family homecare training 15m  S5111 Family homecare train/session  S9482 Family Stabilization 15 Min |
| Psychiatry | 509: Psychiatry- Individual  512: Psychiatry Cons  557: Psychiatry-Group | H0036: Comm Psy Face-Face Per 15min  H0037: Comm Psy Sup Tx Pgm Per Diem  S9480: Intensive Outpatient Psychia  4065F: Antipsychotic pharmacotherapy prescribed (MDD)  80154: Assay Of Benzodiazepines  80156: Assay, Carbamazepine, Total  80164: Assay, Dipropylacetic Acid  80174: Assay Of Imipramine  80178: Assay Of Lithium  80184: Assay Of Phenobarbital  80185: Assay Of Phenytoin, Total  80186: Assay Of Phenytoin, Free  90782: Injection, Sc/Im'  90801: Psy Dx Interview  90862: Medication Management  H2017: Psysoc Rehab Svc, Per 15 Min  J1630: Injection, Haloperidol, Up T  J1631: Injection, Haloperidol Decan  J2680: Injection, Fluphenazine Deca  J2794: Risperidone, Long Acting  T1017: Targeted Case Managemen  J2550: Injection, Promethazine Hcl  J3486: Ziprasidone mesylate  S0163: Injection risperidoneLA  80157: Assay, Carbamazepine, Free |
| Drug and Alcohol | 137: alcohol counsel  507: drug dep-ind  508: alcohol trmt-ind  513: subst abuse-ind  514: subt abuse-home  517: CWT/SUB abuse  518: CWT/TR SUB abuse  519: SUBST/PTSD Teams  555: drug depend-grp  556: alcohol trmt-grp  560: subst abuse-grp  706: alcohol screen | H0047: Alcohol/Drug Abuse Svc Nos  H2034: A/D Halfway House, Per Diem  H2035: A/D Tx Program, Per Hour  H2036: A/D Tx Program, Per Diem  G0396: Alcohol/Subs Interv 15-30mn  G0397: Alcohol/Subs Interv >30 Min  H0049: Alcohol/Drug Screening  H0050: Alcohol/Drug Service 15 Min  H0048: Spec Coll Non-Blood- A/D Test  80100: Drug Screen, Qualitate/Multi  80101: Drug Screen, Single  80102: Drug Confirmation  H0001: Alcohol and/or Drug Assess  H0003: Alcohol and/or Drug Screenin  H0005: Alcohol and/or Drug Services  99408: Alcohol and/or Substance (Other Than Tobacco) Abuse Structured Screening (E.G., Audit, Dast)  99409: Alcohol and/or Substance (Other Than Tobacco) Abuse Structured Screening (E.G., Audit, Dast)  H0002: Alcohol and/or Drug Screenin  H0021: Alcohol and/or Drug Training  82055: Assay Of Ethanol  80103: Tissue Preparation For Drug Analysis  H0004: Alcohol and/or Drug Services  H0006: Alcohol and/or Drug Services  H0007: Alcohol and/or Drug Services  H0008: Alcohol and/or Drug Services  H0009: Alcohol and/or Drug Services  H0010: Alcohol and/or Drug Services  H0011: Alcohol and/or Drug Services  H0012: Alcohol and/or Drug Services  H0013: Alcohol and/or Drug Services  H0014: Alcohol and/or Drug Services  H0015: Alcohol and/or Drug Services  H0016: Alcohol and/or Drug Services  H0017: Alcohol and/or Drug Services  H0018: Alcohol and/or Drug Services  H0019: Alcohol and/or Drug Services  H0020: Alcohol and/or Drug Services  H0022: Alcohol and/or Drug Interven  H0023: Alcohol and/or Drug Outreach  H0024: Alcohol and/or Drug Preventi  H0025: Alcohol and/or Drug Preventi  H0026: Alcohol and/or Drug Preventi  H0027: Alcohol and/or Drug Preventi  H0028: Alcohol and/or Drug Preventi  H0029: Alcohol and/or Drug Preventi  H0030: Alcohol and/or Drug Preventi  T1011: Alcohol/Substance Abuse Noc'  T1012: Alcohol/Substance Abuse Skil  82520: Assay Of Cocaine  80152: Amitriptyline  80154: Benzodiazepines |
